# *Plasmodium falciparum* aldolase and the C-terminal cytoplasmic domain of certain apical organellar proteins promote actin polymerization

**DOI:** 10.1016/j.molbiopara.2014.09.006

**Published:** 2014-10

**Authors:** Suraya A. Diaz, Stephen R. Martin, Munira Grainger, Steven A. Howell, Judith L. Green, Anthony A. Holder

**Affiliations:** a Divisions of Parasitology, MRC National Institute for Medical Research, The Ridgeway, Mill Hill, London NW7 1AA, United Kingdom; b Physical Biochemistry, MRC National Institute for Medical Research, The Ridgeway, Mill Hill, London NW7 1AA, United Kingdom; c Molecular Structure, MRC National Institute for Medical Research, The Ridgeway, Mill Hill, London NW7 1AA, United Kingdom

**Keywords:** Malaria, Merozoite, Motor, Actin, Aldolase, Invasion

## Abstract

The current model of Apicomplexan motility and host cell invasion is that both processes are driven by an actomyosin motor located beneath the plasma membrane, with the force transduced to the outside of the cell *via* coupling through aldolase and the cytoplasmic tail domains (CTDs) of certain type 1 membrane proteins. In *Plasmodium falciparum* (Pf), aldolase is thought to bind to the CTD of members of the thrombospondin-related anonymous protein (TRAP) family, which are micronemal proteins and represented by MTRAP in merozoites. Other type 1 membrane proteins including members of the erythrocyte binding antigen (EBA) and reticulocyte binding protein homologue (RH) protein families, which are also apical organellar proteins, have also been implicated in host cell binding in erythrocyte invasion. However, recent studies with *Toxoplasma gondii* have questioned the importance of aldolase in these processes. Using biolayer interferometry we show that Pf aldolase binds with high affinity to both rabbit and Pf actin, with a similar affinity for filamentous (F-) actin and globular (G-) actin. The interaction between Pf aldolase and merozoite actin was confirmed by co-sedimentation assays. Aldolase binding was shown to promote rabbit actin polymerization indicating that the interaction is more complicated than binding alone. The CTDs of some but not all type 1 membrane proteins also promoted actin polymerization in the absence of aldolase; MTRAP and RH1 CTDs promoted actin polymerization but EBA175 CTD did not. Direct actin polymerization mediated by membrane protein CTDs may contribute to actin recruitment, filament formation and stability during motor assembly, and actin-mediated movement, independent of aldolase.

## 1 Introduction

Like other Apicomplexan parasites, *Plasmodium falciparum* (Pf), a causative agent of malaria has motile forms and can invade host cells. For example, sporozoites are motile and invade hepatocytes, whereas merozoites are not motile but can invade erythrocytes. The merozoite is a polarized cell with specialized subcellular membrane-bound organelles, rhoptries and micronemes, located at the apical end. As found in the other extracellular forms, there is an actomyosin motor located around the periphery of the cell in the space between the surface plasma membrane and flattened alveolar sacs called the inner membrane complex (IMC) that together form the parasite's pellicle. Erythrocyte invasion comprises a series of complex events including low affinity reversible interactions between the surfaces of the two cells, parasite re-orientation to position its apical end closely apposed to the host cell surface, ordered discharge of components from the apical organelles, and then entry of the parasite into a parasitophorous vacuole [1]. Invasion is marked by an annulus or moving junction that travels back over the surface of the parasite as it enters the host cell [2].

In the current model of both invasion and motility [3], largely based on initial studies in another Apicomplexan parasite, *Toxoplasma gondii*, the force required is provided by an actomyosin motor: the myosin is tethered to the IMC as part of the glideosome complex [4] and moves actin filaments to the rear of the cell; these filaments are bound through the tetrameric aldolase molecule to the cytoplasmic tail domains (CTDs) of certain type 1 membrane proteins that transduce the force to the outside of the cell, binding to cell surface receptors or the substratum and pushing the parasite forwards [5]. Although elegant, the correctness of this model for cell invasion has been questioned recently, based on studies in *Toxoplasma gondii* in which the gene for the myosin and other components of the glideosome were deleted [6], and aldolase was shown to be not essential for cell invasion [7].

The merozoite apical organelles contain many proteins, including a number of type 1 membrane proteins such as apical membrane antigen 1 (AMA1) that is proposed to be important in the formation of the moving junction [8], [9], the erythrocyte binding antigen (EBA) and reticulocyte binding protein homologue (RH) protein families that bind to specific receptors on the erythrocyte surface [10], [11], [12], [13], [14], [15], [16], and a member of the thrombospondin repeat anonymous protein (TRAP) family found in merozoites (MTRAP) [17]. In sporozoite stage parasites, TRAP has been identified as the adhesin that couples the movement of actin filaments to the outside of the cell [18]; the TRAP CTD binds to aldolase [4], [17], [19], [20]. In merozoites MTRAP is a micronemal protein that shares key features with TRAP, including a thrombospondin repeat domain, a putative rhomboid-protease cleavage site, and a CTD with a conserved subterminal Trp residue [4], [21]. Some evidence has been provided that MTRAP can interact *in vitro* with aldolase, suggesting that it is the probable merozoite-specific functional homologue of TRAP. However, although MTRAP resembles TRAP structurally and binds to a red cell surface protein [22], its precise role in merozoite invasion of erythrocytes has not been studied in detail. In addition, the CTDs of RH and EBA family proteins have also been shown to bind to aldolase [23]. Although in one previous study Pf aldolase was co-precipitated with F-actin from parasite lysates [24]; the interaction between Pf aldolase and actin has been largely explored using rabbit actin as a surrogate [25], [26], [27]. However, Pf actin is unusual in structure [28], [29] and the experimental use of rabbit actin in its place needs to be validated.

In this study we have investigated the interactions between actin, several CTDs and aldolase. We show that Pf actin and aldolase display high affinity interactions. Furthermore, aldolase promotes the polymerization of rabbit actin, a property it shares with some of the CTDs. The fact that some CTDs alone can stimulate actin polymerization suggests that these CTDs could interact directly with actin in the parasite cytoplasm during motor complex assembly. Overall, these interactions may have a role in the recruitment, polymerization and stabilization of actin, and assembly of the motor complex machinery.

## 2 Materials and methods

### 2.1 Proteins and peptides

#### 2.1.1 Pf aldolase production and validation

The production of GST-tagged Pf aldolase from sequence inserted into pGEX-5X and transformed into BL21 competent cells (Stratagene) has been described previously [25]. Pf aldolase, with the GST tag removed, was purified and its correct folding confirmed by activity measurements in which fructose 1,6-bisphosphate (F1,6P) cleavage was coupled to the triose-phosphateisomerase/α-glycerophosphate dehydrogenase reaction with continuous measurement of NADH consumption monitored at 340 nm (JASCO V550 UV/VIS Spectrophotometer), using an established method [30]. Kinetic analysis was performed using the Lineweaver–Burk plot to calculate the Michaelis–Menten constant (*K*_*m*_) and the maximum reaction velocity (*V*_max_) for the reaction [31].

#### 2.1.2 Purification of actin from *P. falciparum* merozoites

*P. falciparum* 3D7 asexual blood stages were cultured in human red blood cells, and merozoites were purified as described previously [24]. Actin was purified from the cells by extracting globular (G)-actin with 25 mM Tris–HCl (pH 8.0), 0.2 mM CaCl_2_, 0.2 mM ATP, 0.5 mM DTT and clarification by centrifugation at 500,000 × *g* for 20 min as described elsewhere [24]. Filamentous (F)-actin was prepared in actin polymerization buffer (50 mM Tris–HCl (pH 8.0), 500 mM KCl, 20 mM MgCl_2_, 10 mM ATP), and harvested by centrifugation for 20 min at 500,000 *g*. Protein purity was evaluated by SDS-PAGE and Coomassie blue staining.

#### 2.1.3 CTD peptides and rabbit actin

Synthetic peptides based on the sequence of CTDs were purchased from Biomatik (Wilmington, USA): PfMTRAP (PF3D7_1028700, residues 452–498), YFLRKEKTEKVVQEETKEENFEVMFNDDALKGKDNKAMDEEEFWALE; RH1 (PF3D7_0402300, residues 2942–2971), DNNKMDDKSTQKYGRNQEEVMEIFF-DNDYI; and EBA175 (PF3D7_0731500, residues 1445–1502), KYQSSEGVMNENNENNFLFEVTDNLDKLSNMFNQQVQETNINDFSEYHEDINDINFKK. Purified rabbit muscle F- and G-actin were purchased from Sigma–Aldrich. Pyrene-labelled rabbit muscle G-actin, with the fluorescent probe covalently attached to a cysteine side chain [32], was purchased from Cytoskeleton Inc. (Denver, USA).

### 2.2 Protein biotinylation

G- and F-actin from both rabbit and *P. falciparum* were biotinylated using EZ-Link photoactivatable biotin (Pierce, Thermo Scientific) according to the manufacturer's instructions. The nitrophenyl azide biotin compound, which forms a covalent bond with molecules, was incubated with the protein for 5 min at room temperature during exposure to ultraviolet light (350–370 nm). The non-bound biotin was removed by dialysis into 25 mM Tris–HCl (pH 8.0), 0.2 mM CaCl_2_, 0.2 mM ATP, 0.5 mM DTT. Biotinylation was confirmed by biolayer interferometry with streptavidin in the Octet system (see below).

### 2.3 Protein binding assays using biolayer interferometry

The interaction between Pf aldolase and biotinylated G- or F-actin from rabbit and *P. falciparum* was analysed by biolayer interferometry using the Octet Red system (ForteBio) at 25 °C in 96-well microplates. The assays were performed in 100 mM Tris–HCl pH 8.0; all the proteins involved were solubilized in the same buffer using 200 μl per well at the required concentration (0.1–1 μM Pf aldolase) and proceeding as indicated in the manufacturer's instructions. The baseline was established for 20 min and the biotinylated samples were loaded separately onto the streptavidin sensors for 10 min. The sensors were further washed for 5 min and then exposed to Pf aldolase at different concentrations for 20 min, then the dissociation phase was performed for 10 min. The experiments were performed in duplicate or triplicate depending on the experiment. The data were analysed using non-linear least-squares fitting to a 1:1 binding model with in-house software.

### 2.4 Actin co-sedimentation assay

F-actin purified from merozoites was incubated with 6 μg of recombinant Pf aldolase or GST. Following incubation for 20 min at room temperature, the samples were centrifuged for 20 min at 500,000 × *g* to pellet actin filaments. Protein co-sedimentation was evaluated by resolving the proteins in the supernatant and pellet fractions by SDS-PAGE and staining with colloidal Coomassie blue.

### 2.5 The effect of aldolase and CTDs on actin polymerization

Purified pyrene-labelled rabbit G-actin, (Cytoskeleton Inc.) at 0.6 pyrene/monomer equivalence, was prepared at 2.5 μM in 5 mM Tris–HCl pH 8.0, 0.2 mM CaCl_2_. An equal volume of aldolase, CTD, or bovine serum albumin (up to 50 μM final concentration) or no protein was then added prior to polymerization. Actin polymerization was initiated by transfer at a 1 in 10 ratio to 500 mM KCl, 20 mM MgCl_2_, 10 mM ATP according to the manufacturer's instructions, in 10 mm QS cuvettes (Hellma GmbH & Co. KG). Fluorescence measurements were performed in a JASCO FP-6300 spectrofluorimeter at 20 °C and the fluorescence emission intensity recorded for 10 min using an excitation wavelength of 366 nm and an emission wavelength of 407 nm as described previously for pyrene-labelled actin [33]. The rate of actin polymerization was measured as the increase in fluorescence intensity per min. All reactions were carried out in duplicate.

## 3 Results

### 3.1 *P. falciparum* aldolase binds to both rabbit and *P. falciparum* actin

Although previous studies have reported the binding between Pf aldolase and F-actin purified from rabbit muscle [25], we considered it important to establish the equivalence of parasite and rabbit actin, in particular because the structure of the parasite actin differs from that of most higher eukaryotes [28], [29]. Bio-layer interferometry was used to characterize the interaction between catalytically active Pf aldolase and actin from *P. falciparum* or rabbit using streptavidin biosensors loaded with biotinylated G- or F-actin and titration with Pf aldolase concentrations from 0.1 to 1 μM.

**Fig. 1:**
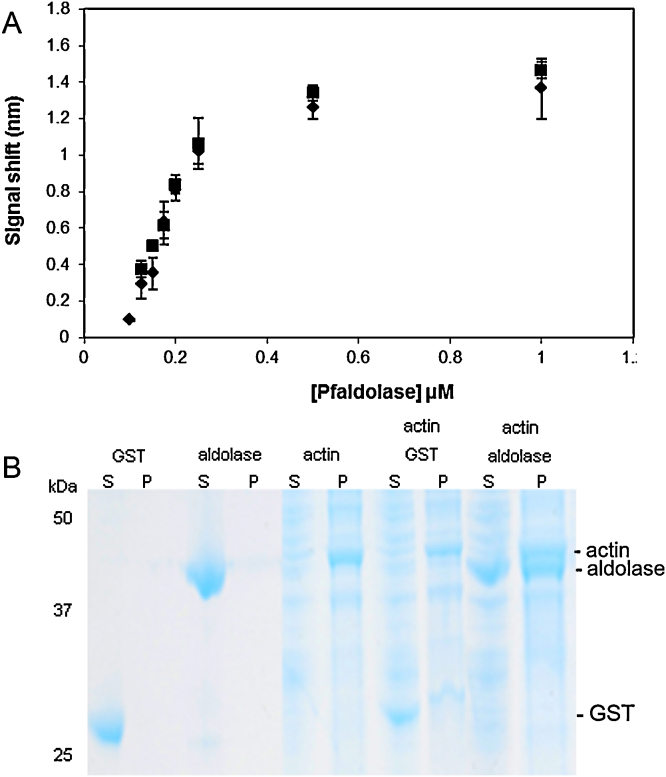
*P. falciparum* aldolase binds to rabbit and parasite actin. (A) Binding to rabbit actin measured using biolayer interferometry. G- (square) or F- (diamond) actin from rabbit muscle was immobilized on the streptavidin coated sensors and aldolase binding was measured at increasing concentrations (0.1–1 μM). The calculated binding constants are given in Table 1. (B) Binding to parasite actin shown by co-sedimentation with merozoite-derived F-actin. Recombinant GST or Pf aldolase were incubated with merozoite F-actin and then supernatant (S) and pellet (P) fractions were obtained following ultracentrifugation. The fractions were resolved by SDS-PAGE and stained with colloidal Coomassie blue. The mobilities of molecular mass markers and actin, aldolase and GST are indicated.

The results for rabbit actin showed high affinity interactions between Pf aldolase and both G- or F-actin with *K*_*d*_s in the high nM range (Fig. 1A and Table 1). F-actin (*K*_*d*_ 365 nM) and G-actin (*K*_*d*_ 330 nM) bound Pf aldolase with similar affinity. The interaction of Pf aldolase with Pf actin was of similar affinity as for rabbit actin, with high affinity interactions in the nM range. In this case, aldolase showed a slightly higher affinity for F-actin (*K*_*d*_ 370 nM) than for G-actin (*K*_*d*_ 470 nM) (Table 1). These results provide the first experimental confirmation of tight binding between parasite aldolase and F-actin and suggest that the results from studies performed with Pf aldolase and rabbit actin can be extrapolated to the interaction between Pf aldolase and Pf actin.

**Table 1:** Dissociation constants for the binding of aldolase to rabbit skeletal or *P. falciparum* actin. The binding assays were performed using ForteBio Octet Red biolayer interferometry with biotinylated actin immobilized on streptavidin coated biosensors, with increasing concentrations of Pf aldolase (0.1–1 μM). The *K*_*d*_ values were calculated from the averaged response from duplicate reactions.

Protein	*K*_*d*_ (μM)	Error
Rabbit G-actin	0.330	0.070
Rabbit F-actin	0.365	0.060
*P. falciparum* G-actin	0.470	0.080
*P. falciparum* F-actin	0.370	0.060

In a separate set of experiments it was shown that recombinant Pf aldolase binds to Pf actin purified from merozoites (Fig. 1B) confirming the interaction. This analysis was performed using co-sedimentation binding assays, which have been widely used in the past to study and analyse the binding between aldolase and muscle F-actin in other species [34], [35], [36], [37]. Analysis of the supernatant and pellet fractions after ultracentrifugation, showed that when incubated in isolation, both recombinant aldolase and the control protein, GST, were present in the supernatant, as expected for soluble proteins. However, when incubated together with F-actin the results showed clearly that aldolase co-sedimented with F-actin in the pellet fraction, whereas GST did not.

### 3.2 Pf aldolase stimulates actin polymerization

**Fig. 2:**
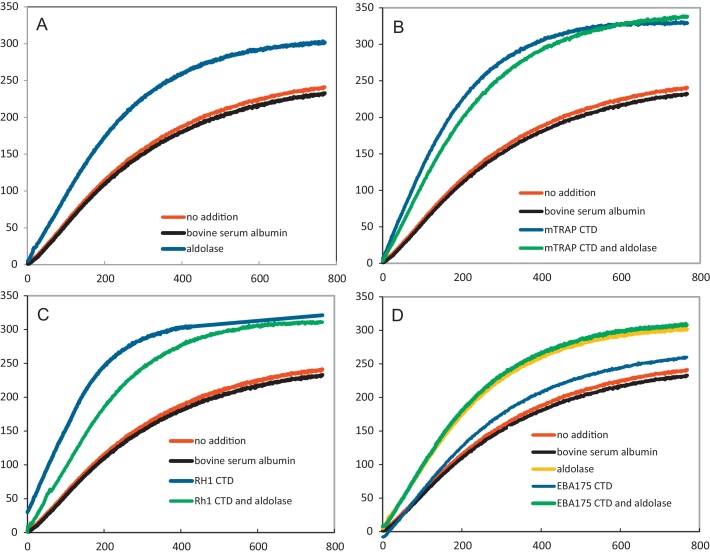
Pf aldolase and sequences from the C-terminal domain (CTD) of apical organellar proteins promote actin polymerization, as measured by fluorescence spectroscopy of pyrene-labelled actin. Actin polymerization was followed in the presence of (A) aldolase; (B) MTRAP CTD and MTRAP CTD and aldolase; (C) RH1 CTD and RH1 CTD and aldolase; and (D) EBA175 CTD, EBA175 CTD and aldolase, and aldolase alone. Polymerization with no addition or in the presence of bovine serum albumin acted as controls for all panels. Protein or peptide was present at 50 μM. Measurements were acquired every second and the means of duplicate readings are presented.

The effect of aldolase on actin polymerization was assessed using pyrene-labelled rabbit skeletal actin, measuring the increase in fluorescence by spectroscopy. The rate and overall level of actin polymerization was stimulated by the presence of aldolase using concentrations between 2.5 and 50 μM (Fig. 2A, Table 2 and data not shown). In contrast the control protein, bovine serum albumin had no effect on either the rate or level of actin polymerization.

**Table 2:** Rates of actin polymerization in the presence of aldolase and CTD peptides. Assays were carried out using 2.5 μM pyrene-labelled rabbit actin together with no addition or in the presence of 50 μM protein/peptide. The data were calculated as the initial rate of fluorescence increase (units min^−1^) from the mean of duplicate reactions.

Protein	Polymerization rate (fluorescence units min^−1)^
Control (no addition)	35
Bovine serum albumin	34
Aldolase	52
MTRAP CTD	66
MTRAP CTD and aldolase	60
RH1 CTD	69
RH1 CTD and aldolase	55
EBA175 CTD	43
EBA175 CTD and aldolase	56

### 3.3 CTD sequences of MTRAP and RH1 but not EBA175 stimulate actin polymerization

Having shown that aldolase can stimulate actin polymerization we asked whether or not the cytoplasmic tails of type 1 membrane proteins implicated in cell invasion might have the same property, especially if aldolase is not essential for coupling actin filaments to cell movement. The fluorescence assay was used to assess the effect of CTD sequences on rabbit actin polymerization, in the presence or absence of aldolase. The results showed that the MTRAP and RH1 CTD sequences increased both the rate and the overall level of actin polymerization (Fig. 2B and C and Table 2). Under the conditions used, the presence of Pf aldolase had no additional effect. In contrast, the EBA175 CTD sequence had no significant effect on actin polymerization (Table 2).

## 4 Discussion

The current model for the *Plasmodium* motor complex includes aldolase and actin as binding partners, allowing the movement of actin to be transduced to the outside of the cell. However, this interaction has been largely studied *in vitro* using actin derived from rabbit muscle and this interaction within the parasite is poorly understood [25], [26], [27], [38]. Aldolase has been identified as an F-actin binding protein [24], but recent studies in *T. gondii* indicate that this interaction is not essential for motility and cell invasion [7]. In the current study we investigated the interaction between malaria parasite aldolase and actin by direct binding and co-sedimentation assays. The direct binding assays indicated that Pf aldolase binds strongly to both rabbit and parasite F- and G-actin with a similar affinity, and recombinant Pf aldolase co-sedimented with parasite F-actin, indicating that aldolase does bind to actin and that the behaviour of rabbit and parasite actin are similar in this regard. These results suggest that the properties of rabbit actin can be extrapolated to *P. falciparum* actin. Overall these results are consistent with the current model in which an interaction between aldolase and actin is central to connecting the motor to drive parasite invasion [17], but do not prove that it is an essential component. Previous reports using F-actin and aldolase from vertebrates and yeast have also indicated a high affinity interaction between these two proteins [35], [36], [39]. These similar observations are consistent with the idea that the binding sites are conserved across organisms from protozoa to humans, as already suggested for vertebrates [35], [36]. Previous reports using F-actin and aldolase isoforms from rabbit, human, fish and yeast had indicated a high affinity interaction between these two protein partners [35], [36], [39].

In addition to binding to rabbit actin Pf aldolase acts to promote its polymerization, increasing both the rate and the final level. This effect was observed at a range of aldolase concentrations up to 50 μM, the highest tested. This concentration is within the physiological intracellular aldolase concentration range, for example in a whole yeast cell the concentration is over 30 μM [40] and the local concentration in the cytosol will greatly exceed this. A similar observation was reported recently using *Babesia* actin [41]. This phenomenon suggests that the formation and assembly of the parasite motor can be stimulated and stabilized by this process. Aldolase might have a role in modulating filament dynamics and motor complex assembly, consistent with a previous report that aldolase stimulates actin polymerization and cytoskeleton assembly in guinea pig sperm [42]. Previously it has been proposed that other proteins such as profilin and formins are necessary for actin filament formation and it cannot be ruled out that such additional cofactors are also important *in vivo* [4].

Surprisingly, it was found that sequences derived from the CTDs of certain type 1 membrane proteins implicated in cell invasion, also increased both the rate and the final level of actin polymerization. For example, MTRAP and RH1 but not EBA175 sequences were effective. Under the conditions used the presence or absence of aldolase had no additional effect on the ability of these sequences to polymerize actin. This suggests that these CTDs can interact directly with actin in the parasite cytoplasm to promote motor complex assembly through actin filament formation and stabilization, possibly recruiting actin to the motor complex. Alternatively, these CTDs may interact with actin at the outer surface of micronemes, before discharge. These results also provide an explanation of why aldolase may not be required or essential for motor function and raise the possibility that assembly and polymerization of actin filaments by these organellar proteins is a key process in cell invasion.

In summary, *P. falciparum* aldolase can bind with high affinity to actin, and one result of this binding is an increase in the rate and level of actin polymerization. In addition, even in the absence of aldolase the CTD of some type 1 organellar proteins such as MTRAP and RH1 can also stimulate actin polymerization. This ability to stimulate actin polymerization suggests that these proteins have a key role in actin filament dynamics, motor complex assembly and stability, and cell invasion.

## Competing interests

The authors declare that they have no competing interests.
